# Supplementary figures and images for: Pupillary response is associated with the reset and switching of functional brain networks during salience processing
Source: PLoS Comput Biol. 2023 May 12;19(5):e1011081. doi: 10.1371/journal.pcbi.1011081 (PMC10208478; doi:10.1371/journal.pcbi.1011081)

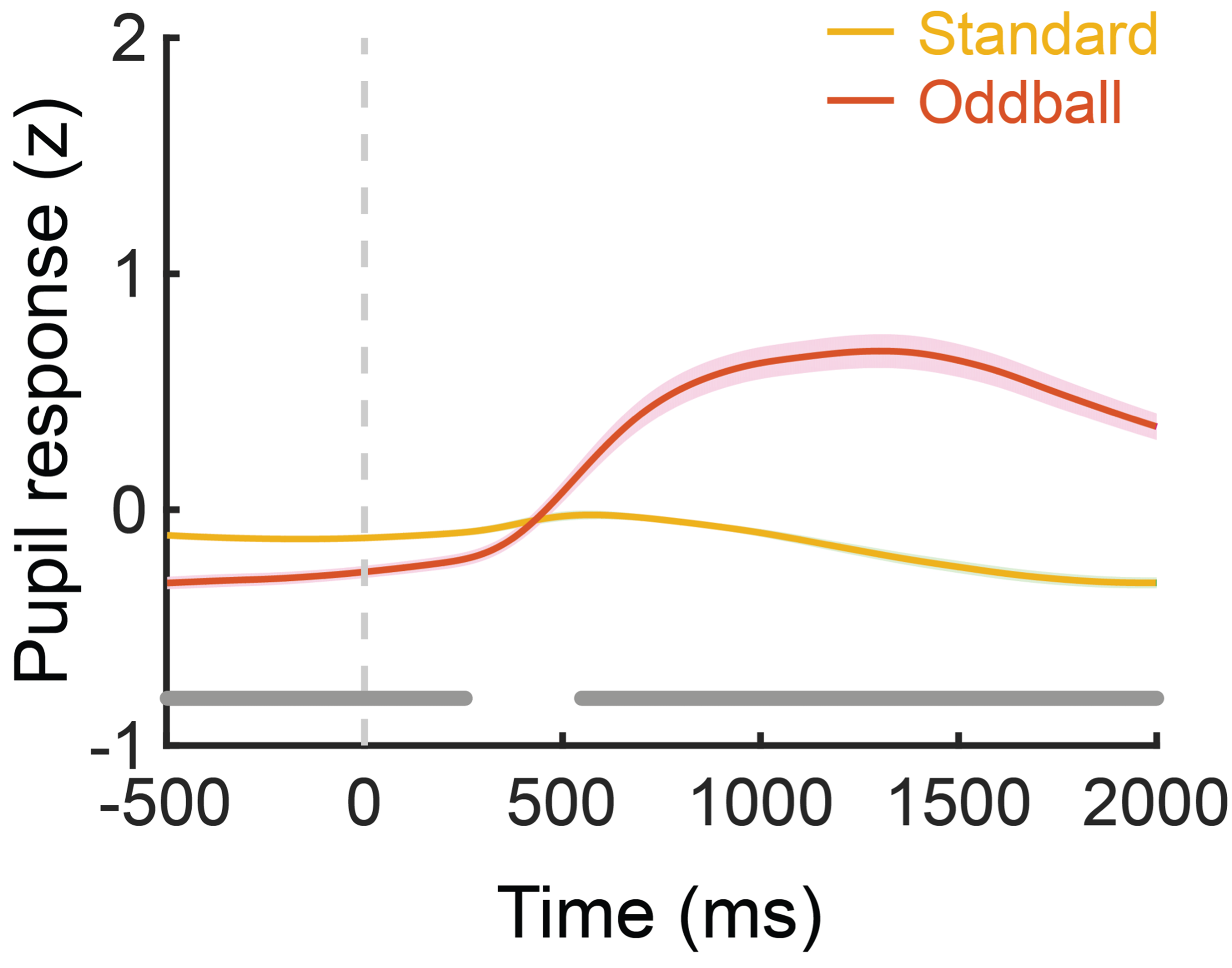

Supplement: S1 Fig — The z-scored pupil diameter fluctuations from 500 ms before the stimulus to 2000 ms following the stimulus were averaged across subjects for the oddball (red) and standard (yellow) stimuli. The shaded bands represent standard error, and the bottom gray line indicates significant difference (Student’s t-test, p < 0.001) between the pupil diameter evoked by the oddball and standard stimuli. (TIF) [file pcbi.1011081.s001.tif]

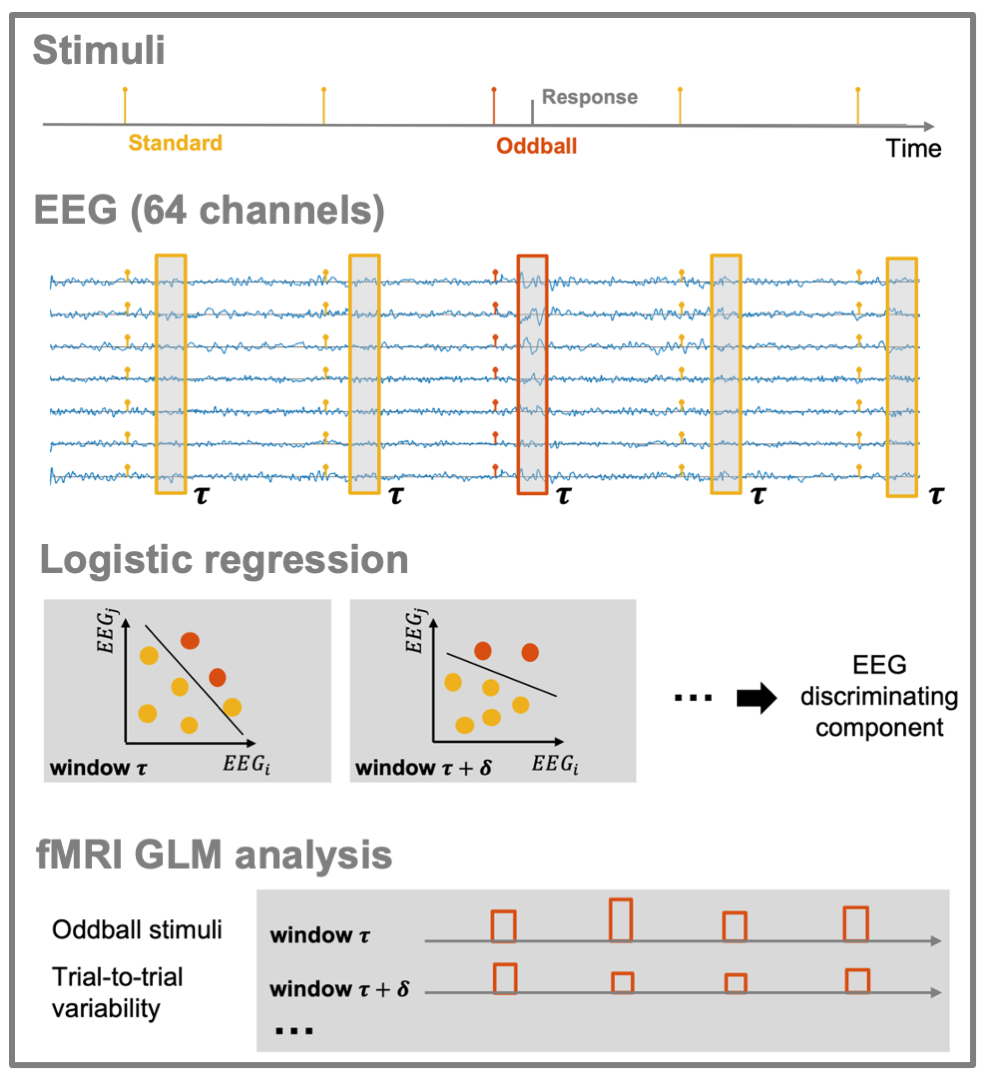

Supplement: S2 Fig — For each temporal window τ, we applied a single-trial analysis with the extracted EEG data in the windows from all the trials, where a logistic regressor was trained to learn a weight matrix w maximally discriminating the target vs standard trials. From the weighting on the EEG channels with matrix w, a EEG discriminating component was computed as a low-dimensional representation of the EEG data. For example, two EEG sensors (channel i and j) were illustrated in the figure with a hyperplane discriminating target (red dots) and standard (yellow dots) trials. Similarly, single-trial analysis was applied to all other temporal windows spanning the trial independently with a sliding window approach (step size as δ). The EEG discriminating component at each temporal window was used to modulate regressors in a general linear model (GLM) to predict fMRI BOLD response (convolved with the canonical hemodynamic response function along with other regressors). The GLM analysis was applied with each temporal window independently. (TIF) [file pcbi.1011081.s002.tif]

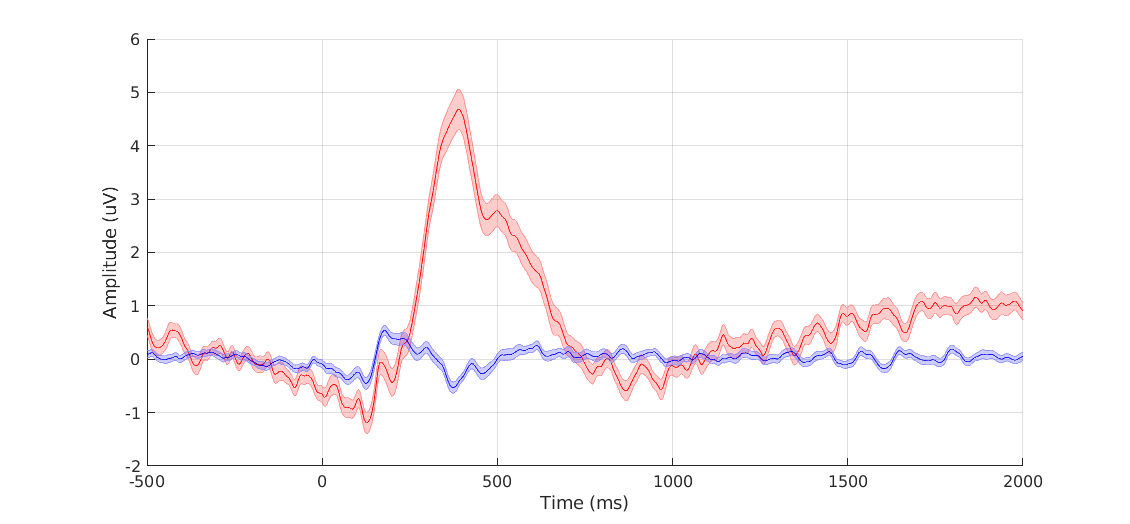

Supplement: S3 Fig — The solid lines denote the group mean, and the shaded areas denote the standard error across subjects. The P300 component was observed with a peak around 390 ms. (TIF) [file pcbi.1011081.s003.tif]

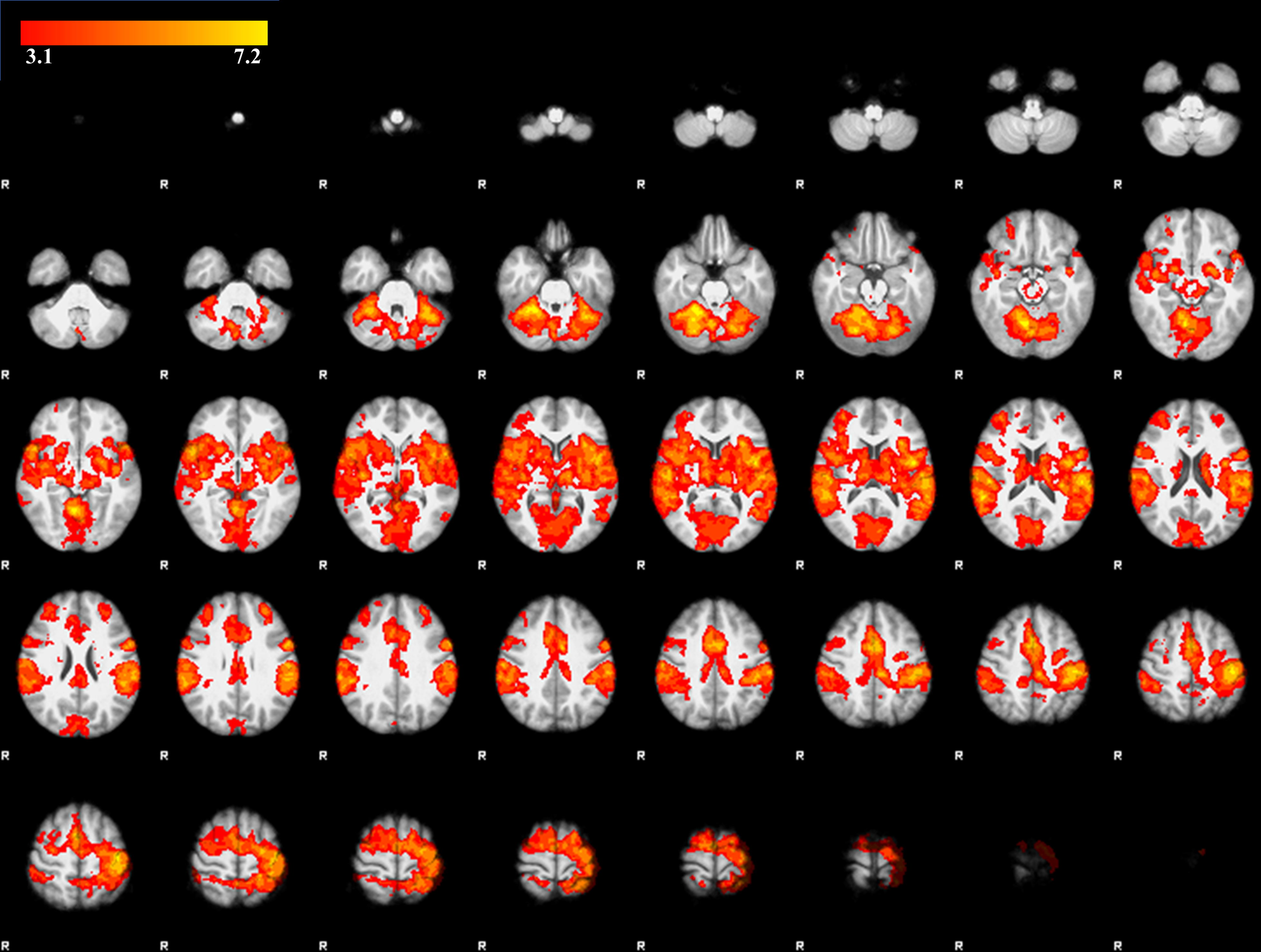

Supplement: S4 Fig — The z-statistic maps were displayed on top of the MNI152 template brain image. FMRIB’s Local Analysis of Mixed Effects (FLAME) from the FSL software package was used for the group-level statistical inference. The group-level statistical parametric maps were thresholded with z > 3.1 and corrected cluster significance threshold of p = 0.05 (Gaussian random field method). Regions in the dorsal attention network, salience network, visual and auditory cortex, primary somatosensory cortex, and subcortex were identified as significant clusters. Please be noted that only the significant positive effects are shown here, and we did not observe any significant negative effects in the regions of the default mode network. The ‘R’ in the figure denotes right side of the brain. (TIF) [file pcbi.1011081.s004.tif]

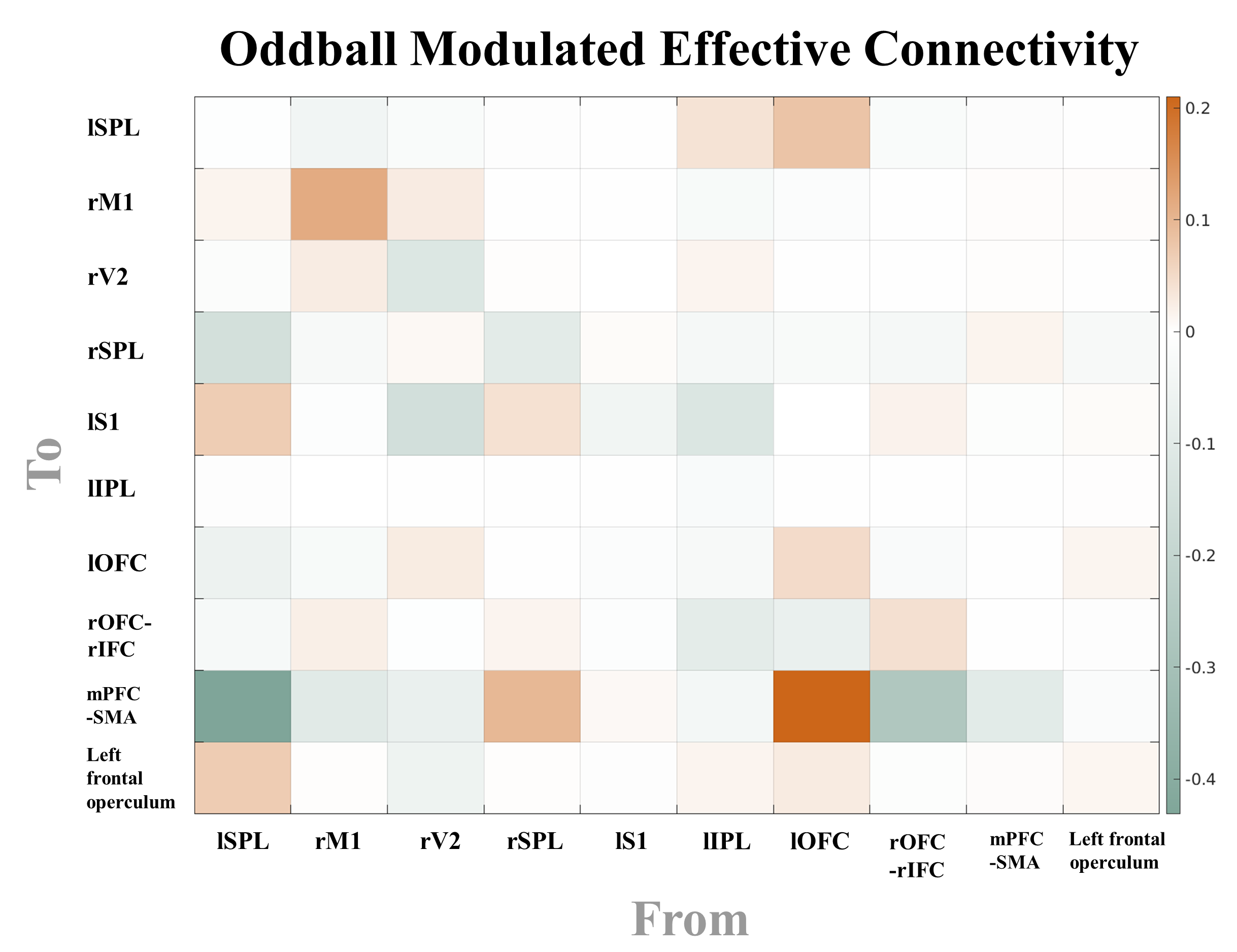

Supplement: S5 Fig — Please be noted the results here reflect mean group effect. The orange and blue color represents positive and negative effective connectivity, respectively. (TIF) [file pcbi.1011081.s005.tif]

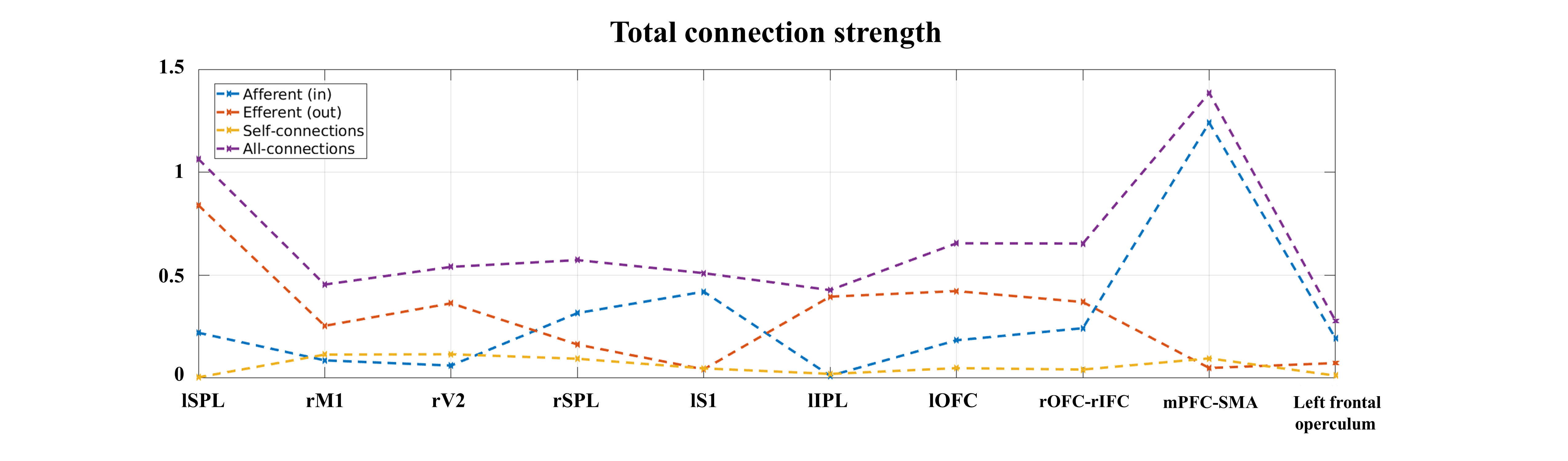

Supplement: S6 Fig — With the effective connectivity results, all the unsigned connection parameters (efferent, afferent and self-connection) associated with the node were summed up to compute the total connection strength. The results suggest that the lSPL and mPFC-SMA have the strongest total connection strength, indicating their roles as hubs in the processing of salience stimuli. (TIF) [file pcbi.1011081.s006.tif]

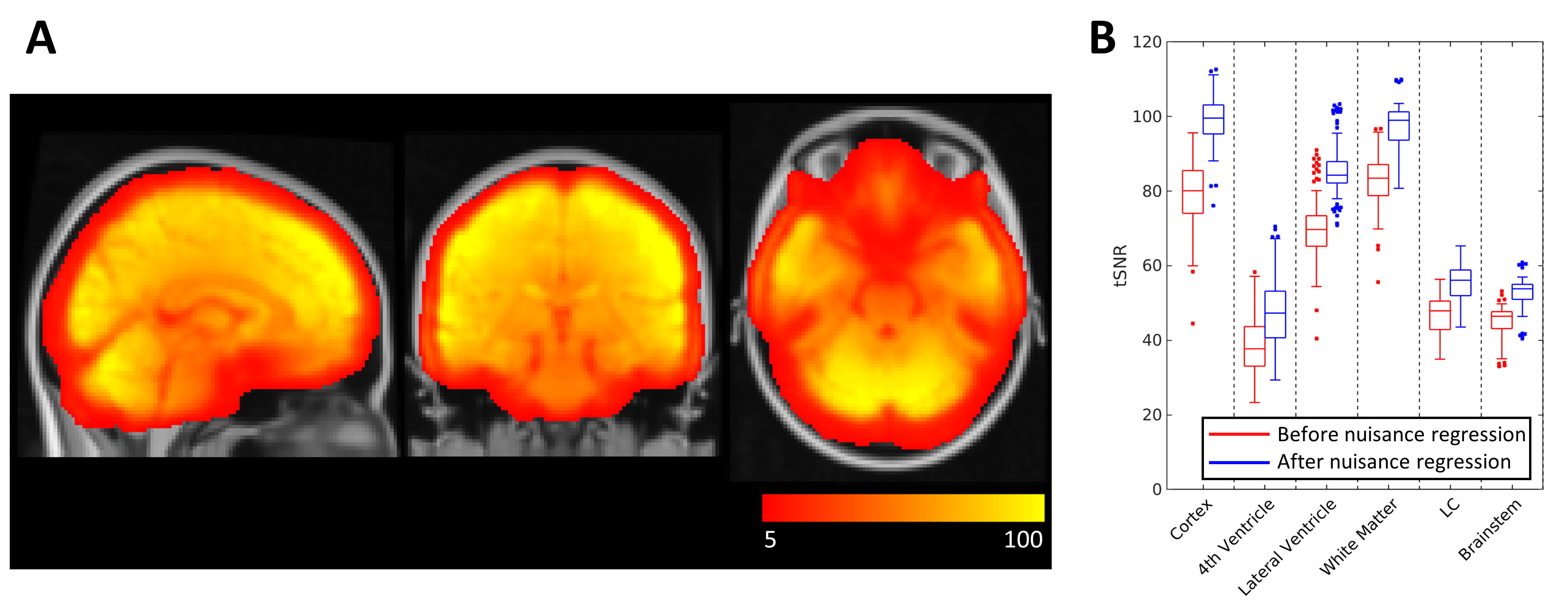

Supplement: S7 Fig — The tSNR was computed for each voxel, by dividing the mean over the standard deviation. (A) Group-level mean tSNR map of preprocessed fMRI data (no spatial smoothing). The tSNR map of each run was spatially normalized into the MNI152 space, and then was averaged across all the runs of subjects. (B) Quantitative analysis and boxplot of tSNR distributions across runs in each ROI. The color denotes the tSNR before (red) and after (blue) the nuisance signal regression (motion parameters and BOLD signals in the 4th ventricle and the left and right hemisphere white matter and lateral ventricles). Before the functional connectivity analysis of the LC, we regressed out the BOLD signal in the 4th ventricle. The tSNR was computed for each voxel in the subject’s native functional space, and then was averaged within the ROI (segmented with FreeSurfer). The LC two standard deviation template was used to delineate the LC ROI [91]. The tSNR in the LC is above the standard cut-offs (tSNR > 30) [92]. (TIF) [file pcbi.1011081.s007.tif]

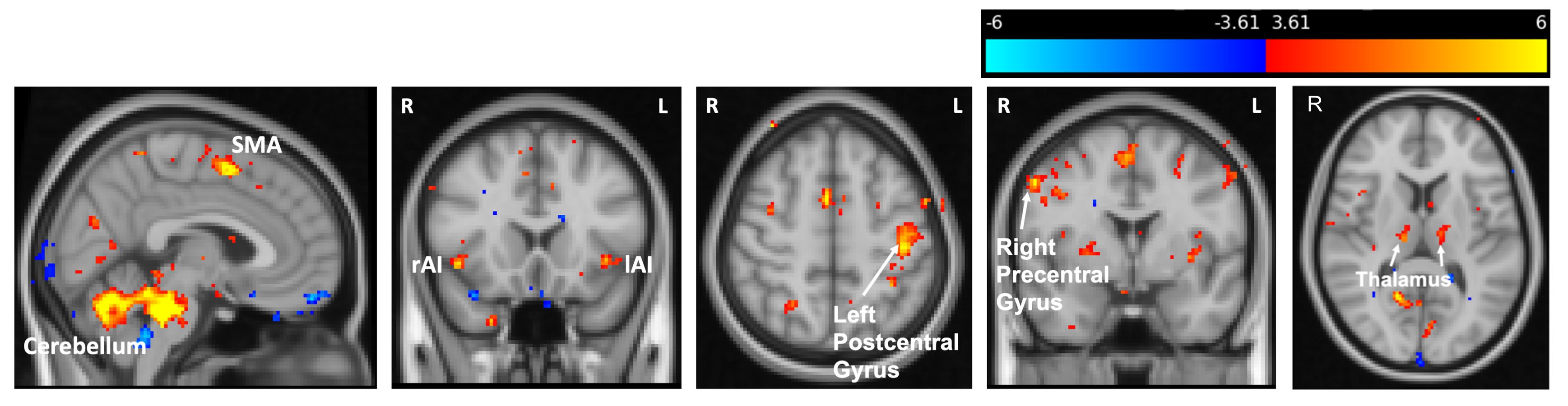

Supplement: S8 Fig — We used a mixed effects model for group inference. Each subject’s FC map was transformed into z-score with Fisher’s Z transformation. And the FC z-score map was thresholded at p < 0.01. In the group-level, one sample student’s t-test was performed to obtain the significant seed-based FC map of the LC (p < 0.001 uncorrected). Significant clusters were identified in the cerebellum, supplementary motor area (SMA), right and left anterior insula (AI), left postcentral gyrus, right precentral gyrus, and thalamus. (TIF) [file pcbi.1011081.s008.tif]

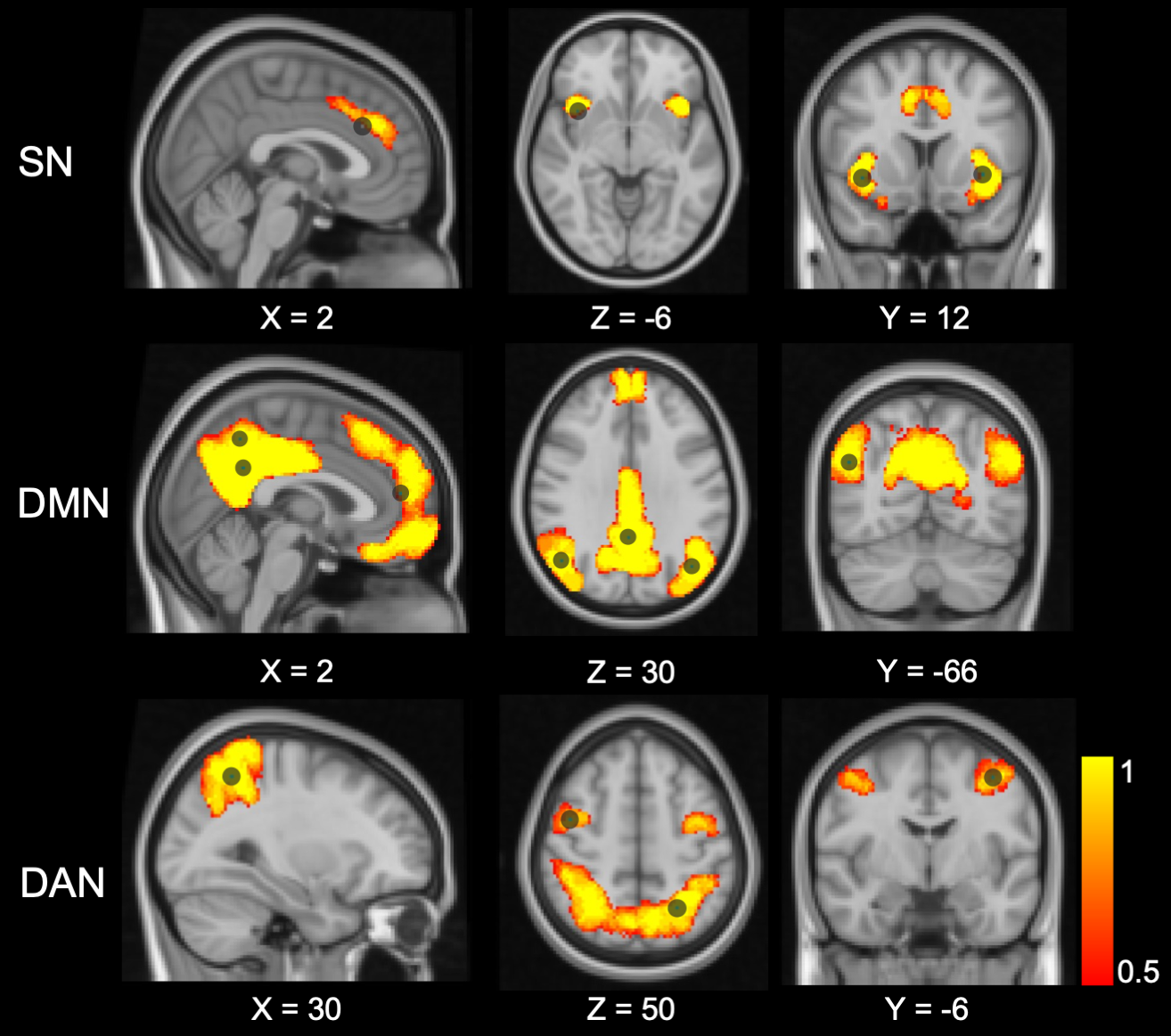

Supplement: S9 Fig — The nodes (circles) of the SN, DMN, and DAN are overlaid with the selected network areas from the HCP-MMP atlas and the MNI152 brain image. The group-level region of interest masks (illustrated as spatial distribution maps) were obtained from majority vote across subjects. (TIF) [file pcbi.1011081.s009.tif]

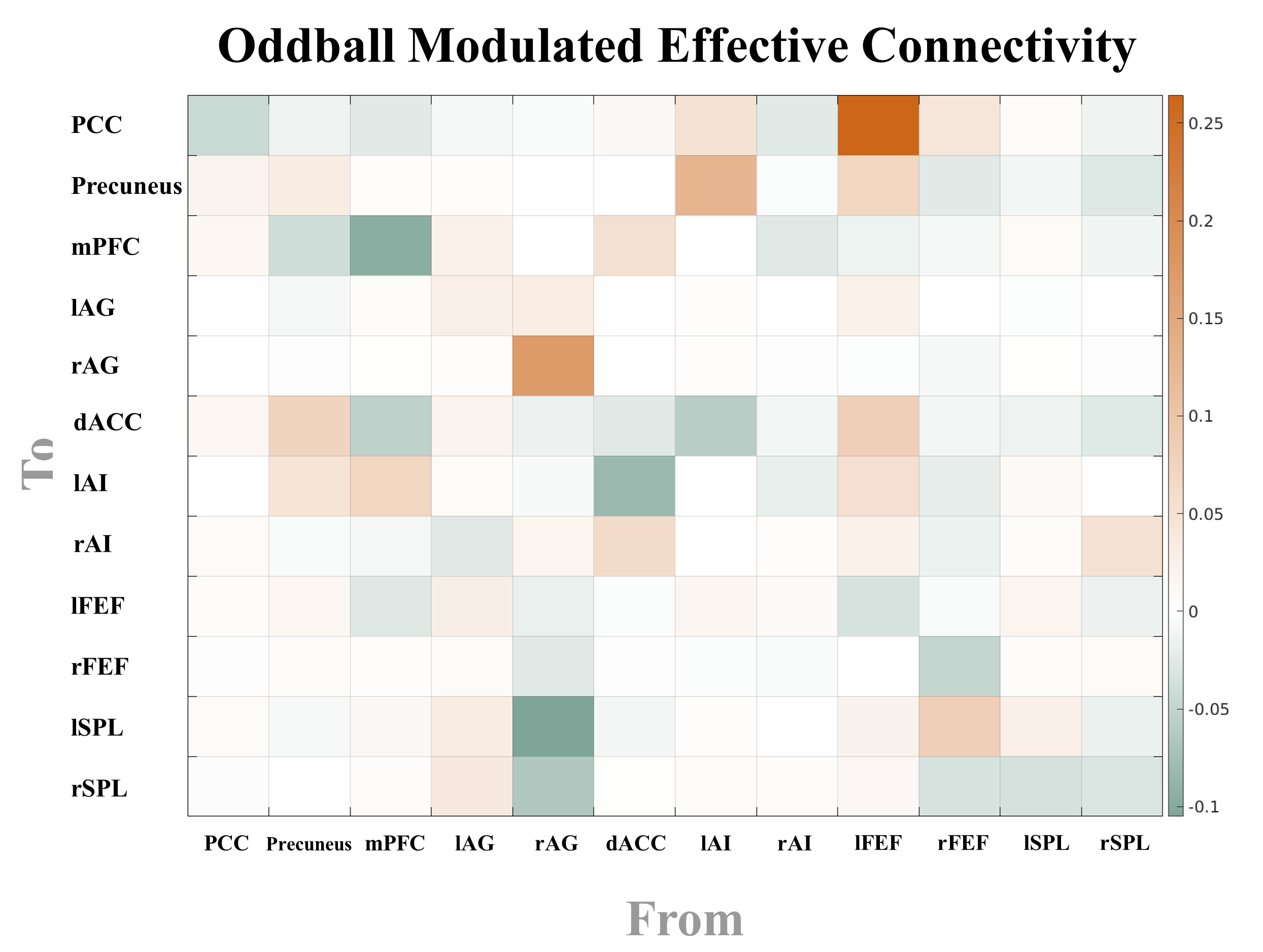

Supplement: S10 Fig — The orange and blue color represents positive and negative effective connectivity, respectively. (TIF) [file pcbi.1011081.s010.tif]

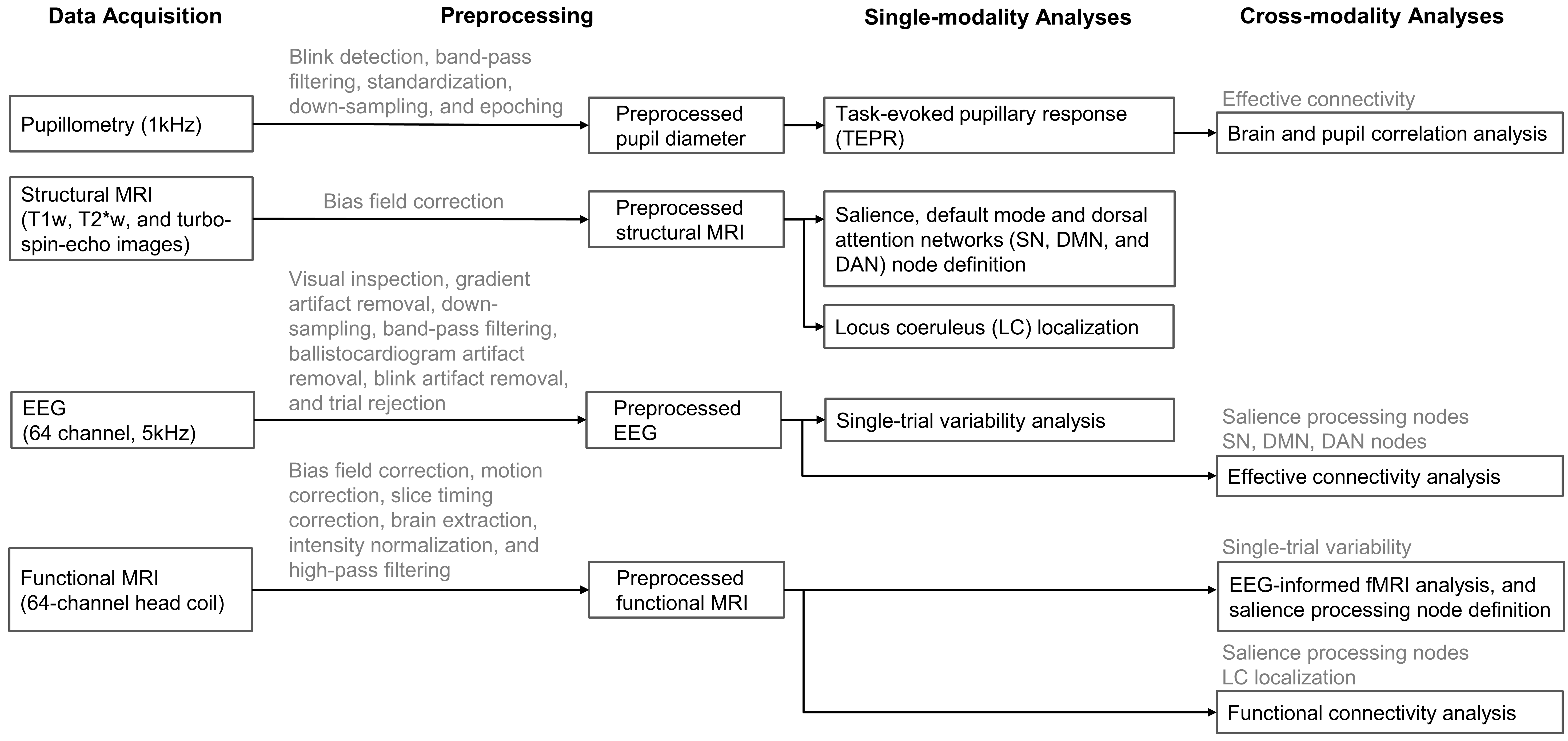

Supplement: S11 Fig — (TIF) [file pcbi.1011081.s011.tif]
