# Supplementary material for: Pupillary response is associated with the reset and switching of functional brain networks during salience processing
Source: PLoS Comput Biol. 2023 May 12;19(5):e1011081. doi: 10.1371/journal.pcbi.1011081 (PMC10208478; doi:10.1371/journal.pcbi.1011081)
Supplement: S1 Text — We provide additional descriptions on data preprocessing, network node definition, locus coeruleus localization, and additional results on brain-pupil relationships. (PDF) [file pcbi.1011081.s012.pdf]

Supplementary Materials for

Pupillary response is associated with the reset and switching of functional brain networks during salience processing

Hengda He*, Linbi Hong, Paul Sajda*

*Corresponding author. hengda.he@columbia.edu (H.H.); psajda@columbia.edu (P.S.)

This PDF file includes:

S1 Text
References

S1 Text

Pupillometry data preprocessing and epoching

The preprocessing pipeline was adapted from the approach in [1]. Firstly, blink detection was performed with Eyelink software, and then the blinks were padded by 150 ms and linearly interpolated. Additional blinks were further removed with a peak detection algorithm. We computed pupil diameter from the pupil area data, and then the pupil diameter time series were filtered with a bandpass second-order Butterworth filter (0.01 Hz to 10 Hz). Then, the pupil diameter data of each run were z-scored independently, and down-sampled to 500 Hz (the same sampling rate of the preprocessed EEG data). The preprocessed pupil diameter data were epoched from 500 ms before the stimulus to 2000 ms following the stimulus. Two pupil diameter measurements were examed including the prestimulus baseline pupil diameter (BPD) and the task-evoked pupillary response (TEPR). The BPD was defined as the averaged pupil diameter from 500 ms before the stimulus to the onset of the stimulus, and TEPR was defined as the maximum percentage deviation from BPD within each epoch.

EEG preprocessing

Visual inspection was first performed, to make sure the raw EEG signal was not contaminated by confounding factors, including TR volume jitter and data saturation. An average artifact template subtraction approach was then used to remove the gradient artifact with Brain Products' Analyzer2 data processing software [2], [3]. The data were then down-sampled to 500 Hz. After the gradient artifact removal, a tenth order median filter was applied, to reject any residual gradient artifact. Then, the EEG data were filtered with a fourth order bandpass Butterworth filter (0.5 Hz to 50 Hz), to remove DC drift and high frequency noise. After filtering, each subject's EEG data were concatenated over runs for the application of ballistocardiogram artifact (BCG) removal. Specifically, QRS detection was first carried out, and then the BCG was removed with EEGLAB's FMRIB plugin (simple mean approach). The BCG removed data were then re-referenced to the common average. The final step of preprocessing is blink artifact removal, where independent component analysis (ICA) was performed using EEGLAB's ICA function to compute ICs, manually identify and remove the blink ICs [4]. The preprocessed EEG data were epoched identically as the pupillometry data from 500 ms before stimulus to 2000 ms following the stimulus. Then, baseline correction was carried out by removing the mean baseline value, which was computed from 500 ms before the stimulus onset to the stimulus onset for each epoch. We performed a trial rejection using a probability distribution based criteria. Specifically, we rejected trials where the EEG signal from a single channel that is outside of 6SD, and the EEG signals from all channels that are outside of 2SD. We also rejected trials where subjects incorrectly responded or failed to respond.

Structural and functional MRI preprocessing

The fMRI data were processed using FSL (V6.0) [5]. Briefly, motion correction was performed using rigid-body registrations on all the volumes in reference to the middle time point volume [6]. Then, slice timing correction was carried out with Fourier-space time-series phase-shifting. The non-brain tissues were removed using BET [7]. After that, grand-mean intensity normalization was applied by scaling the entire 4D data with a multiplicative factor. Lastly, a high-pass filtering (Gaussian-weighted least-squares straight line fitting, cut-off frequency 0.01 Hz) was carried out. No global signal regression was applied, since the global signal includes neuronal-related signals, and global signal regression has shown to introduce artificial negative

correlations in the functional connectivity [8]. In the spatial normalization, the middle time point EPI volume head image of the fMRI data was rigid registered (cost function as correlation) to the high resolution T2*w head image. Then, the T2*w head image was rigid registered (Boundary-based method) [9] to the T1w head image. Lastly, the subject's T1w brain image was initially affine transformed and then non-linearly registered to the MNI152 (the nonlinear 6th generation atlas from FSL) brain image using FLIRT [6] and FNIRT [10] from the FSL software package. The structural T1w images were processed using FreeSurfer pipeline [11], which resulted in brain tissue segmentations and surfaces reconstruction. The FreeSurfer segmentations of the T1w images include gray matter, white matter, lateral ventricles, brain masks, etc. And each subject's T1w image was also used to construct the volumetric head model and the source model using the FieldTrip toolbox [12] for the effective connectivity state-space model.

Salience processing node definition

The oddball EEG STV related GLM statistical maps at each time of interest from the STV EEG-informed fMRI analysis were extracted for defining the nodes associated with the processing of oddball trials. At the peak voxel of each group-level significant cluster, a spherical region of interest (ROI) (10 mm radius) centered on the voxel was generated. Centroid of peak locations was used for regions involved in more than one temporal windows (i.e. lS1, rSPL, and mPFC-SMA). After excluding one cluster with the peak voxel outside of the brain mask, ten ROIs were included for the subsequent analyses. The neuroanatomical localizations of the nodes were referred to the Human Connectome Project Multi-Modal Parcellation (HCP-MMP) [13]. Specifically, to alleviate inter-subject variability in the cortical surface reconstruction, instead of mapping the HCP-MMP cortex parcellation to the volumetric space, the ROI masks in each subject's native structural space were projected to the subject's cortical surface. After surface-based spatial normalization to the FreeSurfer fsaverage template cortical surface [14], a majority vote was caried out across subjects to obtain the group-level ROI surface areas, which were compared to the HCP-MMP parcellation for the naming of the anatomical locations of the nodes.

SN, DMN, and DAN node definition

The locations of the nodes in the salience network (SN), default mode network (DMN), and dorsal attention network (DAN) were defined with the HCP-MMP atlas. The SN comprised three nodes: right and left anterior insula (AI) (each side includes area anterior agranular insular complex and middle insular area) and dorsal anterior cingulate cortex (ACC) (includes area dorsal 32, anterior 32 prime, and p32 prime; details of area naming in [13]). The DMN comprised five nodes: posterior cingulate cortex (includes RSC, 23d, 23c, d23ab, v23ab, 31a, 31pv, 31pd, POS1, POS2, 7m, DVT, ProS), precuneus (includes PCV), right and left angular gyrus (each side includes PGi, PGs, and PGp), and medial prefrontal cortex (includes 8BM, 9m, 10r, and 10v). The DAN comprised four nodes: right and left SPL (each side includes LIPv, LIPd, VIP, AIP, MIP, 7PC, 7AL, 7Am, 7PL, 7Pm, IP0, IP1, and IP2), and right and left frontal eye fields. The HCP-MMP atlas was transformed to each subject's cortical surface through surface-based registration using FreeSurfer. Then, the selected ROI surface areas were projected into the volumetric space. The ROI masks were warped into the MNI152 template space with the previously estimated registration parameters. A majority vote was caried out across subjects to obtain the group-level ROI masks. After thresholding the ROI masks (0.5 overlap rate across subjects), we extracted a weighted center of gravity for each ROI region. Similar to the previous analysis, a spherical ROI (10 mm radius) centered on that voxel was generated. Finally, these

twelve nodes were used for the subsequent EEG effective connectivity state-space modeling. Details of the SN, DMN, and DAN nodes are illustrated in the Figure supplement 9.

Locus coeruleus localization and fMRI analysis

To assess functional connectivity between the locus coeruleus (LC) and salience processing nodes, we localized the LC in each subject's functional space with a predefined LC atlas [15] and the subject's turbo spin echo (TSE) image, and then the LC BOLD signal was extracted. Specifically, we first performed a rough localization by estimating the spatial range of the LC location in each subject's structural T1-weighted space, where a statistical criterion was used along with the TSE image intensity spatial distribution and the LC atlas. Then, the TSE image intensity within the estimated range was transformed into the subject's functional EPI space for a precise localization of the LC. The LC BOLD signal was extracted by averaging the voxel-wise BOLD time series, weighted by the TSE image intensities. Details are in [16]. Functional connectivity was computed with Pearson correlation between the BOLD signals extracted from the LC and salience processing nodes (mixed effect, $p < 0.05$ uncorrected). The LC showed significant functional connectivity with lSPL, lS1 and mPFC-SMA (lSPL: $t = 2.64$, $p = 0.017$; lS1: $t = 3.80$, $p = 0.001$; mPFC-SMA: $t = 3.15$, $p = 0.006$), however, there were no significant results between the LC and the other salience processing nodes (rM1: $t = 0.73$, $p = 0.476$; rV2: $t = -1.15$, $p = 0.267$; rSPL: $t = 1.48$, $p = 0.156$; lIPL: $t = -0.45$, $p = 0.660$; lOFC: $t = -0.56$, $p = 0.579$; rOFC-rIFC: $t = -1.92$, $p = 0.071$; Left frontal operculum: $t = 0.96$, $p = 0.351$).

Relationship between pupillary response and effective connectivity of salience processing early, middle, and late time networks

We evaluated the association between the late-to-early positive network strength and TEPR by computing the Pearson correlation between them. The significance level was set as $\alpha < 0.05$ with Bonferroni correction. For the positive connections, whilst the late-to-early network strength showed a significant correlation with TEPR ($r = 0.6352$, $p = 0.0035$), the other network connectivity strength did not show any statistically significant relationship with TEPR (Early-to-early: $r = 0.4290$, $p = 0.0668$; Early-to-middle: $r = 0.3708$, $p = 0.1181$; Early-to-late: $r = 0.0916$, $p = 0.7091$; Middle-to-early: $r = 0.1163$, $p = 0.6354$; Middle-to-middle: $r = 0.1219$, $p = 0.6191$; Middle-to-late: $r = 0.1058$, $p = 0.6665$; Late-to-middle: $r = 0.1404$, $p = 0.5665$; Late-to-late: $r = -0.1568$, $p = 0.5214$). For the negative connections, none of these network connections strength showed significant correlation with TEPR.

Relationship between pupillary response and effective connectivity of SN, DAN, and DMN

To test our hypothesis that the LC is associated with the network switching function of the SN, we computed the Pearson correlation of TEPR with SN-to-DAN positive network EC strength and SN-to-DMN negative network EC strength (SN-to-DAN positive: $r = 0.6804$, $p = 0.0013$; SN-to-DMN negative: $r = -0.6055$, $p = 0.0060$), which aligns with the direction of information flow between SN, DAN, and DMN in the literature. However, to fully eliminate the possibly alternative models that differ in the direction of information flow between SN, DAN, and DMN, we performed the same analysis and evaluated the association between the TEPR and the inter-network effective connectivity between SN, DAN, and DMN. The significance level was set as $\alpha < 0.05$ with Bonferroni correction. For the positive connections, whilst the SN-to-DAN network strength showed a significant correlation with TEPR, the other network connectivity strength did not show any statistically significant relationship with TEPR (DAN-to-DMN: $r = -0.2710$, p =

0.2616; DAN-to-SN: r = -0.0800, p = 0.7447; DMN-to-DAN: r = 0.4827, p = 0.0363; DMN-to-SN: r = 0.0570, p = 0.8167; SN-to-DMN: r = 0.3780, p = 0.1106). For the negative connections, whilst the SN-to-DMN network strength showed a significant correlation with TEPR, the other network connectivity strength did not show any statistically significant relationship with TEPR (DAN-to-DMN: r = 0.0244, p = 0.9209; DAN-to-SN: r = 0.0864, p = 0.7248; DMN-to-DAN: r = -0.4895, p = 0.0334; DMN-to-SN: r = -0.2279, p = 0.3478; SN-to-DAN: r = -0.2849, p = 0.2370).

References

[1] A. E. Urai, A. Braun, and T. H. Donner, “Pupil-linked arousal is driven by decision uncertainty and alters serial choice bias,” *Nat. Commun.*, vol. 8, no. 1, pp. 1–11, 2017.

[2] P. J. Allen, O. Josephs, and R. Turner, “A method for removing imaging artifact from continuous EEG recorded during functional MRI,” *Neuroimage*, vol. 12, no. 2, pp. 230–239, 2000.

[3] R. Abreu, A. Leal, and P. Figueiredo, “EEG-informed fMRI: A review of data analysis methods,” *Frontiers in Human Neuroscience*, vol. 12. Frontiers Media S. A, p. 29, Feb. 06, 2018, doi: 10.3389/fnhum.2018.00029.

[4] T.-P. Jung, S. Makeig, M. Westerfield, J. Townsend, E. Courchesne, and T. J. Sejnowski, “Removal of eye activity artifacts from visual event-related potentials in normal and clinical subjects,” *Clin. Neurophysiol.*, vol. 111, no. 10, pp. 1745–1758, 2000.

[5] S. M. Smith *et al.*, “Advances in functional and structural MR image analysis and implementation as FSL,” *Neuroimage*, vol. 23, pp. S208–S219, 2004, doi: 10.1016/j.neuroimage.2004.07.051.

[6] M. Jenkinson, P. Bannister, M. Brady, and S. Smith, “Improved optimization for the robust and accurate linear registration and motion correction of brain images,” *Neuroimage*, vol. 17, no. 2, pp. 825–841, 2002.

[7] S. M. Smith, “Fast robust automated brain extraction,” *Hum. Brain Mapp.*, vol. 17, no. 3, pp. 143–155, 2002.

[8] C. Caballero-Gaudes and R. C. Reynolds, “Methods for cleaning the BOLD fMRI signal,” *Neuroimage*, vol. 154, pp. 128–149, Jul. 2017, doi: 10.1016/J.NEUROIMAGE.2016.12.018.

[9] D. N. Greve and B. Fischl, “Accurate and robust brain image alignment using boundary-based registration,” *Neuroimage*, vol. 48, no. 1, pp. 63–72, 2009.

[10] J. L. R. Andersson, M. Jenkinson, S. Smith, and others, “Non-linear registration, aka Spatial normalisation FMRIB technical report TR07JA2,” *FMRIB Anal. Gr. Univ. Oxford*, vol. 2, no. 1, p. e21, 2007.

[11] B. Fischl *et al.*, “Automatically parcellating the human cerebral cortex,” *Cereb. Cortex*, vol. 14, no. 1, pp. 11–22, Jan. 2004, doi: 10.1093/cercor/bhg087.

[12] R. Oostenveld, P. Fries, E. Maris, and J. M. Schoffelen, “FieldTrip: Open source software for advanced analysis of MEG, EEG, and invasive electrophysiological data,” *Comput. Intell. Neurosci.*, vol. 2011, 2011, doi: 10.1155/2011/156869.

[13] M. F. Glasser *et al.*, “A multi-modal parcellation of human cerebral cortex,” *Nature*, vol. 536, no. 7615, pp. 171–178, Aug. 2016, doi: 10.1038/nature18933.

[14] B. Fischl, M. I. Sereno, R. B. H. H. Tootell, and A. M. Dale, “High-resolution intersubject averaging and a coordinate system for the cortical surface,” *Hum. Brain Mapp.*, vol. 8, no. 4, pp. 272–284, 1999, doi: 10.1002/(SICI)1097-0193(1999)8:4<272::AID-HBM10>3.0.CO;2-4.

[15] N. I. Keren, C. T. Lozar, K. C. Harris, P. S. Morgan, and M. A. Eckert, “In vivo mapping of the human locus coeruleus,” *Neuroimage*, vol. 47, no. 4, pp. 1261–1267, Oct. 2009, doi: 10.1016/j.neuroimage.2009.06.012.

[16] H. He, L. Hong, and P. Sajda, “An automatic and subject-specific method for locus coeruleus localization and BOLD activity extraction,” *Proc Int Soc Magn Reson Med*, 2021.
