# Supplementary material for: Pupillary response is associated with the reset and switching of functional brain networks during salience processing
Source: PLoS Comput Biol. 2023 May 12;19(5):e1011081. doi: 10.1371/journal.pcbi.1011081 (PMC10208478; doi:10.1371/journal.pcbi.1011081)
Supplement: Attachment — Submitted filename: Responses-to-reviewers.docx [file pcbi.1011081.s013.docx]

**Reviewers’ Comments to the authors:**

**Reviewer #1:**

This is an interesting and comprehensive multimodal study of the salience network and its relationship to LC. The data and results would be of interest to the readers in the neuroscience community.

Major comment

The data and results presented are quite extensive and interesting, the presentation could be a bit clearer. First, it would be useful to add the chart to illustrate all the steps of the data analyses.

Also, it would be useful to add a summary of the results/conclusion at the end.

It is not clear how the 5 statistical parametric maps obtained with EEG-STV regression analysis of the fMRI are combined and/or used.

Minor comment

I would change "a fMRI" to "an fMRI" on page 5.

**Reviewer #2:**

He et al. report an EEG-fMRI-pupillometry study involving an auditory oddball task in 19 healthy adults. They report a variety of analyses centered on interactions between the LC-NE system and networks including the SN, DAN and DMN. The analyses are rigorous, and the EEG-informed fMRI approach adds important temporal information about network activations during oddball events. The effective connectivity findings provide interesting (though somewhat difficult to interpret) preliminary findings, and the authors acknowledge limitations of that approach. Overall, this a technically impressive multimodal study that has the potential to advance understanding of interactions between neuromodulatory systems (LC-NE) and intrinsic brain networks in relation to cognition. To enhance the impact of the study, my comments center on scholarship, better situating the results within existing literature, clarifications, and some potential inconsistencies with prior findings.

- fMRI research on networks that respond to multimodal (e.g. auditory/visual/somatosensory) salience was conducted well before many of the papers cited by the authors. For example, see Downar et al. (Nat Neurosci 2000; J Neurophysiol 2002) which shows involvement of salience network regions prior to the “salience network” terminology was introduced, using paradigms similar to the one used here. I suggest including these earlier references and discussing in light of the present findings.

- Deactivations in the salience network in response to oddball events were very surprising, given what is usually observed (however, DMN deactivations would be expected). The supplement shows standard GLM results that do seem to show (expected) salience network activations, but the EEG-informed fMRI analyses suggest deactivations. What explains this discrepancy, and were DMN deactivations found in the standard GLM analysis (only activations are shown)?

- The results in figure 5 are based on individual differences and the sample size is small, raising questions about power and statistical bias. To support these findings, could the authors look at intra-individual, trial-by-trial relationships (which would be well powered and would still address the question of interest here)?

- The final section of Results focus on the LC, but the second half of that section focused only on pupillary responses (TEPR) as an index of the LC. The authors did examine LC activity for functional connectivity analyses but it does not appear that they looked at LC phasic responses and their relationships with pupillary responses and cortical networks. Rather than using TEPR as an index of LC here, why not look at LC activity itself?

- Several intracranial EEG studies have been mapping out the temporal dynamics of task-evoked responses in DAN, SN, and DMN (Raccah et al 2018 J Neurosci; Kucyi et al 2020 Nat Commun) as well as relationships between the SN and pupillary responses (Kucyi et al 2020 J Neurosci). Such findings also highlight early DAN responses (prior to SN) and SN-pupil relationships potentially implicating the LC-NE system. The discussions in this paper, while rigorous in terms of fMRI and EEG/MEG literature, have not included these very (likely even more) relevant references that relate strongly to the present findings. How do these findings build on such prior intracranial EEG work?

- The discussion section on broader implications of pupillometry-EEG-fMRI could better acknowledge prior studies that have also used this valuable approach (e.g. Groot et al., 2021 Neuroimage).

- More data acquisition specifications for EEG conducted with fMRI are needed in the Methods section. For example, was the MR clock synchronized to EEG recordings to reduce noise? How were the electrode impedance levels set and tested prior to subjects entering the scanner?

**PCOMPBIOL-D-22-01699: Response to reviewers**

Dear editors and reviewers,

We thank the reviewers for their constructive comments and the positive assessment of our work. We have clarified the methodology and included more discussion with respect to the existing literature. Please find below our point-by-point reply (in black) to the reviewers’ comments (printed in *blue italics*), and the modified text (printed in red) in the revised manuscript.

We hope that you will now find our manuscript suitable for publication in *PLOS Computational Biology*.

Sincerely,

Hengda He, Linbi Hong, and Paul Sajda

*Reviewer #1: This is an interesting and comprehensive multimodal study of the salience network and its relationship to LC. The data and results would be of interest to the readers in the neuroscience community.*

We thank the reviewers for their supportive comments. Please find our replies to your specific comments below.

*Major comment

The data and results presented are quite extensive and interesting, the presentation could be a bit clearer. First, it would be useful to add the chart to illustrate all the steps of the data analyses.*

Thank you for this suggestion. We have now included a chart summarizing all the data processing steps in Supplementary Figure 11. We also added the following text to the beginning of the methods section.

In this section, we describe the analyses of the multimodal pupillometry-EEG-fMRI data, specifically focused on experimental design, data acquisition and preprocessing, EEG single-trial analysis, EEG-informed fMRI analysis, fMRI functional connectivity analysis, EEG effective connectivity analysis, and brain-pupil correlation analysis. A flow chart is included in S11 Fig. to illustrate the steps of data processing and single-modality/cross-modality data analyses.

S11 Fig. Flow chart illustrating the steps of data acquisition, preprocessing, single-modality analyses, and cross-modality analyses.

*Also, it would be useful to add a summary of the results/conclusion at the end.*

We have added a summary of the results/conclusion as the Conclusions section in the manuscript, please see below.

Conclusions

In summary, the present multimodal study analysis of pupillometry-EEG-fMRI reveals a coordinated neural cascade during salience processing, involving brain regions in the dorsal attention, visual, motor, executive control, and salience networks. Additionally, the study identified the network organization and effective connectivity between these brain regions, which were found to be associated with salient stimuli evoked pupillary response. These findings are in line with the proposed function of the pupil-indexed LC-NE system in network reset (Bouret et al., 2005; Corbetta et al. 2008). To advance understanding of the interactions between neuromodulatory systems and intrinsic brain networks in relation to cognition, the study presented additional analyses on the associations between the LC-NE system and networks including the SN, DAN, and DMN, where the results provide the first evidence in humans for the relevance of the LC-NE system to the function of the SN in the dynamic switching between anticorrelated cortical networks. This work has important implications for multimodal neuroimaging data analyses, brain-pupil relationships, attentional processing, cognitive control networks, and network models of neurological diseases.

*It is not clear how the 5 statistical parametric maps obtained with EEG-STV regression analysis of the fMRI are combined and/or used.*

Thanks for pointing this out. We added a sentence at the end of the method section “STV EEG-informed fMRI analysis” to clarify this issue.

As the oddball EEG STV regressor was the primary regressor of interest (i.e., the regressor indicative of task-relevant processing), we only used cortical regions whose BOLD signal covaries with this particular regressor in the subsequent analyses.

*Minor comment

I would change "a fMRI" to "an fMRI" on page 5.*

This was fixed in the revised manuscript.

*Reviewer #2: He et al. report an EEG-fMRI-pupillometry study involving an auditory oddball task in 19 healthy adults. They report a variety of analyses centered on interactions between the LC-NE system and networks including the SN, DAN and DMN. The analyses are rigorous, and the EEG-informed fMRI approach adds important temporal information about network activations during oddball events. The effective connectivity findings provide interesting (though somewhat difficult to interpret) preliminary findings, and the authors acknowledge limitations of that approach. Overall, this a technically impressive multimodal study that has the potential to advance understanding of interactions between neuromodulatory systems (LC-NE) and intrinsic brain networks in relation to cognition. To enhance the impact of the study, my comments center on scholarship, better situating the results within existing literature, clarifications, and some potential inconsistencies with prior findings.*

Thank you for the supportive comments. We are pleased that you find our work “technically impressive” and will be of use in guiding future research. Please find our replies to your specific comments below.

*- fMRI research on networks that respond to multimodal (e.g. auditory/visual/somatosensory) salience was conducted well before many of the papers cited by the authors. For example, see Downar et al. (Nat Neurosci 2000; J Neurophysiol 2002) which shows involvement of salience network regions prior to the “salience network” terminology was introduced, using paradigms similar to the one used here. I suggest including these earlier references and discussing in light of the present findings.*

We thank the reviewers for directing us to this literature. We have included this literature when discussing the brain regions involved in the processing of salient stimuli. We also included a discussion of these references in light of our findings in the discussion section, please see below.

The involvement of SN/M-CIN regions in processing salient stimuli was reported by (Downar et al., 2000 & 2002), where the anterior cingulate and the supplementary motor areas had been proposed a role in anticipation of forthcoming stimuli.

*- Deactivations in the salience network in response to oddball events were very surprising, given what is usually observed (however, DMN deactivations would be expected). The supplement shows standard GLM results that do seem to show (expected) salience network activations, but the EEG-informed fMRI analyses suggest deactivations. What explains this discrepancy, and were DMN deactivations found in the standard GLM analysis (only activations are shown)?*

Thank you for this valuable comment. A short answer to the question is that the standard GLM results in the supplementary material represent mean effects across the trials, whereas the EEG-informed fMRI analysis results represent the effects of trial-to-trial variability. Specifically, in the standard GLM analysis, the regressor of interest is the contrast between the regressors of the oddball and standard trials, where the trials/events in each regressor were constructed with the same unmodulated height. Thus, the results in the supplementary material, showing significant activations of the salience network, indicate that the mean response of this network is stronger for the oddball trials compared to the standard trials. Whereas, these two regressors were included as nuisance regressors in the EEG-informed fMRI analysis, where we are interested in a height-modulated regressor of oddball trials. The height difference between trials represents the discriminating (oddball v.s. standard trials) component extracted from the EEG signal. Therefore, the results, showing a negative activation of the salience network in the late window, indicate that this network responds stronger when an oddball trial is less discriminating from the standard trials in the late window.

However, we agree with the reviewer that these results seem to be inconsistent at the first glance, and we agree that the negative sign in the salience network responses is hard to interpret. We have included the following sentence in the discussion to clarify this issue.

~~The present findings are also in line with the proposed "windshield wiper" mechanism (Sadaghiani et al., 2015) of the SN.~~ It is worth noting that, in the traditional fMRI analysis of oddball effects (Fig. S4), regions in the SN were identified as significantly activated clusters, whereas, our single-trial variability EEG-informed fMRI analysis results suggest that the SN was deactivated in response to oddball events in the late window (Fig. 1&6). These observations indicate that though the mean response of the SN is stronger for the oddball trials compared to the standard trials, our EEG-informed time-resolved analysis shows that late in the trial, when an oddball is less discriminating from a standard, as measured by EEG, the SN response is stronger to the oddball trial. We believe this result supports the proposed "windshield wiper" role of the SN in the literature (Sadaghiani et al., 2015), and a stronger response in the SN might suppress the discriminating processes of the current trial, facilitating task disengagement. However, this hypothesis warrants further investigation.

Thanks for pointing out the ambiguity in the supplementary figure (Fig. S4) of the standard GLM analysis. This figure shows the significant effects only on the positive side (oddball v.s. standard events). We did not observe any significant negative effects in the DMN areas. This is consistent with fMRI research literature using oddball tasks, for example, in a meta-analysis of visual and auditory oddball effect studies (Kim, 2014), they also did not observe any significant negative effects in the DMN. As many studies have reported that the strength of DMN deactivations scales with task complexity, cognitive demand, and task difficulty, it is expected that we did not observe any significant DMN effects in such a simple task (auditory oddball). We have added a sentence to the supplementary figure (Fig. S4) caption of standard GLM analysis to clarify this ambiguity.

Kim H. Involvement of the dorsal and ventral attention networks in oddball stimulus processing: A meta-analysis. Human Brain Mapping. 2014;35(5):2265–2284. doi:10.1002/HBM.22326.

Please be noted that only the significant positive effects are shown here, and we did not observe any significant negative effects in the regions of the default mode network.

*- The results in figure 5 are based on individual differences and the sample size is small, raising questions about power and statistical bias. To support these findings, could the authors look at intra-individual, trial-by-trial relationships (which would be well powered and would still address the question of interest here)?*

We agree with the reviewer that this additional intra-individual analysis will provide better power and would be an interesting experiment. However, this trial-by-trial analysis requires an estimation of the effective connectivity parameters for each trial, which is very challenging. Based on our previous experiments, if we fit the state-space effective connectivity model separately for the EEG signals of each trial segment, the estimation of the parameters will be biased by the quality of model fitting. Therefore, we adopted the approach to fit the model for the EEG signals of each run, examine the brain-pupil relationship at an inter-individual level, and we showed that these results (as in figure 5) are not biased by the quality of model fitting. The reviewer proposed an interesting experiment to test our findings at an intra-individual level, which could be assessed in the future when more sessions/acquisitions are available for each individual. We have added the following sentences to the discussion section to address this concern.

In this study, we examined the relationship between effective connectivity and pupil measurement at the inter-individual level as shown in Fig. 5. Future studies are needed to replicate our results at the intra-individual level. However, care should be taken when fitting the effective connectivity model without enough data points. And more experimental sessions and data acquisition for each individual may be needed.

*- The final section of Results focus on the LC, but the second half of that section focused only on pupillary responses (TEPR) as an index of the LC. The authors did examine LC activity for functional connectivity analyses but it does not appear that they looked at LC phasic responses and their relationships with pupillary responses and cortical networks. Rather than using TEPR as an index of LC here, why not look at LC activity itself?*

Thank you for this valuable comment. Our results showed significant functional connectivity between the LC and salience processing nodes in the dorsal attention and salience networks (DAN and SN). This aligns with the literature showing the spatial specificity in the projections of LC neurons throughout the cortex. Intriguing, this might explain our findings that the pupil-indexed LC activity is associated with the network reset signal from the SN to DAN. However, we agreed with the reviewer that it would be more interesting to look at LC activity itself. This shortcoming of the manuscript and the challenges in neuroimaging of the LC were discussed in the Limitations section of the manuscript. Additional analyses of the LC BOLD activity and its relationship to the cortex processes and pupillary responses were performed and described elsewhere, see abstract and manuscript (Hong et al., 2022; He et al., 2021). We have added a sentence in the Limitations section to highlight and direct readers to these additional results.

Additionally, the present study did not examine the relationship between the LC activity and pupillary response, as well as its relationship to the EEG measurement of cortex processes. Another study from our group highlighted these additional analyses and comprehensive examinations of LC activity and their associations (Hong et al., 2022; He et al., 2021).

Hong L, He H, Sajda P. Pupil-linked phasic arousal relates to the reduction of response inhibition: inferences from a simultaneous pupillometry and EEG-fMRI study. bioRxiv. 2022; p. 2022–08

He H, Hong L, Sajda P. An Automatic and Subject-specific Method for Locus
Coeruleus Localization and BOLD Activity Extraction. Proc Int Soc Magn Reson
Med. 2021;29:2711.

*- Several intracranial EEG studies have been mapping out the temporal dynamics of task-evoked responses in DAN, SN, and DMN (Raccah et al 2018 J Neurosci; Kucyi et al 2020 Nat Commun) as well as relationships between the SN and pupillary responses (Kucyi et al 2020 J Neurosci). Such findings also highlight early DAN responses (prior to SN) and SN-pupil relationships potentially implicating the LC-NE system. The discussions in this paper, while rigorous in terms of fMRI and EEG/MEG literature, have not included these very (likely even more) relevant references that relate strongly to the present findings. How do these findings build on such prior intracranial EEG work?*

We thank the reviewers for directing us to this literature. We have included a discussion on the relevance between our findings and the results in these intracranial EEG studies. In summary, our results in this study and a previous report from our group align with these intracranial EEG studies’ findings, regarding the temporal profiles in the task responses of DAN, SN, and DMN. The following discussions on these intracranial EEG studies have been added to the discussion section.

~~The single-trial EEG-informed fMRI analysis results suggest a late involvement of the SN areas and an early involvement of DAN areas, however, these results did not provide information on the interactions among them.~~ Regarding the temporal profiles of the task responses in SN, DAN, and DMN, our results align with several intracranial EEG studies (Raccah et al 2018 J Neurosci; Kucyi et al 2020 Nat Commun), where they found the responses reached the fastest, intermediate, and slowest speed in the DAN, SN, and DMN, respectively. In our study, we also found an earlier involvement of the DAN (starting at 225 ms), preceding the responses in the SN starting at 400 ms. Interestingly, in our previous simultaneous EEG-fMRI study (Walz et al., 2014), we identified significant responses in the DMN at a relatively late time window (525 ms), supporting the temporal order of responses in DAN, SN, and DMN as reported in these intracranial EEG studies. However, these findings in the task-evoked responses do not imply interactions among these networks. In this study, our effective connectivity analyses extend such frameworks of networks' temporal dynamics, by proposing that the network switching signal from the SN to the DAN might reflect a late-to-early feedback signal.

In an intracranial EEG study (Kucyi et al., 2020), both the spontaneous and task-evoked activations in the anterior insula are linked to the dynamics of pupillary dilation.

Kucyi A, Parvizi J. Pupillary dynamics link spontaneous and task-evoked activations recorded directly from human insula. Journal of Neuroscience. 2020;40(32):6207–6218.

Raccah O, Daitch AL, Kucyi A, Parvizi J. Direct cortical recordings suggest temporal order of task-evoked responses in human dorsal attention and default networks. Journal of Neuroscience. 2018;38(48):10305–10313.

Kucyi A, Daitch A, Raccah O, Zhao B, Zhang C, Esterman M, et al. Electrophysiological dynamics of antagonistic brain networks reflect attentional fluctuations. Nature communications. 2020;11(1):325.

Walz JM, Goldman RI, Carapezza M, Muraskin J, Brown TR, Sajda P. Simultaneous EEG-fMRI reveals a temporal cascade of task-related and default-mode activations during a simple target detection task. NeuroImage. 2014;102(P1):229–239. doi:10.1016/j.neuroimage.2013.08.014.

*- The discussion section on broader implications of pupillometry-EEG-fMRI could better acknowledge prior studies that have also used this valuable approach (e.g. Groot et al., 2021 Neuroimage).*

We agreed with the reviewer. Therefore, we have expanded the discussion section on the previous two studies (Mayeli et al., 2020; and Groot et al., 2021) which made use of simultaneous acquisition of pupillometry, EEG, and fMRI. Please see below.

In addition to the other work from our group where we used this multi-modal approach to examine the relationship between LC activity, pupillary response, and cortical dynamics (Hong et al., 2022), there are two other very recent instances where this simultaneous triple-modality data acquisition was reported (Mayeli et al., 2020; Groot et al., 2021). Together with the work presented here, all four studies showcase unique analyses and insights that could be harnessed from simultaneous pupillometry-EEG-fMRI. For instance, while this work and Hong et al. used an asymmetric fusion approach, Groot et al. applied a symmetric fusion to the multi-modal data through a support vector machine, in order to investigate neural signatures of task-unrelated thoughts. Taken together, we believe that despite the technical challenges, this simultaneous multi-modal approach holds great value and potential in unraveling cortical dynamics at various levels.

Mayeli A, Al Zoubi O, Misaki M, Stewart JL, Zotev V, Luo Q, et al. Integration of simultaneous resting-state electroencephalography, functional magnetic resonance imaging, and eye-tracker methods to determine and verify electroencephalography vigilance measure. Brain Connectivity. 2020;10(10):535–546.

Groot JM, Boayue NM, Csifcs ́ak G, Boekel W, Huster R, Forstmann BU, et al. Probing the neural signature of mind wandering with simultaneous fMRI-EEG and pupillometry. NeuroImage. 2021;224:117412.

*- More data acquisition specifications for EEG conducted with fMRI are needed in the Methods section. For example, was the MR clock synchronized to EEG recordings to reduce noise? How were the electrode impedance levels set and tested prior to subjects entering the scanner?*

The reviewer raised a great point here. It was one of the co-first authors (Linbi Hong) who developed the experimental protocol and acquired data. The data acquisition specifications (along with the rigorous measures taken to ensure data quality) were described in great detail in Hong et al., 2022, which the readers will be directed. We added the following sentences in the Methods section to refer the readers to this information.

Data acquisition specifications (along with the rigorous measures taken to ensure data quality) were described in detail in Hong et al., 2022. Specifically, proper synchronization between EEG recording and MR imaging was ensured via Brain Products' SyncBox. The SyncBox receives pulses coming from the scanner's gradient clock board directly, and can therefore synchronize the sampling rate of the amplifier with the scanner clock system (Brain-Products, 2019). And to ensure subject safety during simultaneous EEG and fMRI acquisition, the scalp and ECG electrodes were embedded with series resistors of 10 kOhm and 20 kOhm, respectively. During the experiment, electrodes’ impedances were kept under 25 kOhm (including the built-in resistors on each electrode) to minimize the noise in EEG acquisition.

Brain-Products. Brainamp series and brainamp mr series operating instructions. Brain Products GmbH. 2019. Available from: [https://www.brainproducts.com/downloads/manuals/brainamp-series-brainamp-mr-series-operating-instructions](https://www.brainproducts.com/downloads/manuals/brainamp-series-brainamp-mr-series-operating-instructions/)
